# Supplementary figures and images for: Does perioperative respiratory event increase length of hospital stay and hospital cost in pediatric ambulatory surgery?
Source: PLoS One. 2021 May 13;16(5):e0251433. doi: 10.1371/journal.pone.0251433 (PMC8118274; doi:10.1371/journal.pone.0251433)

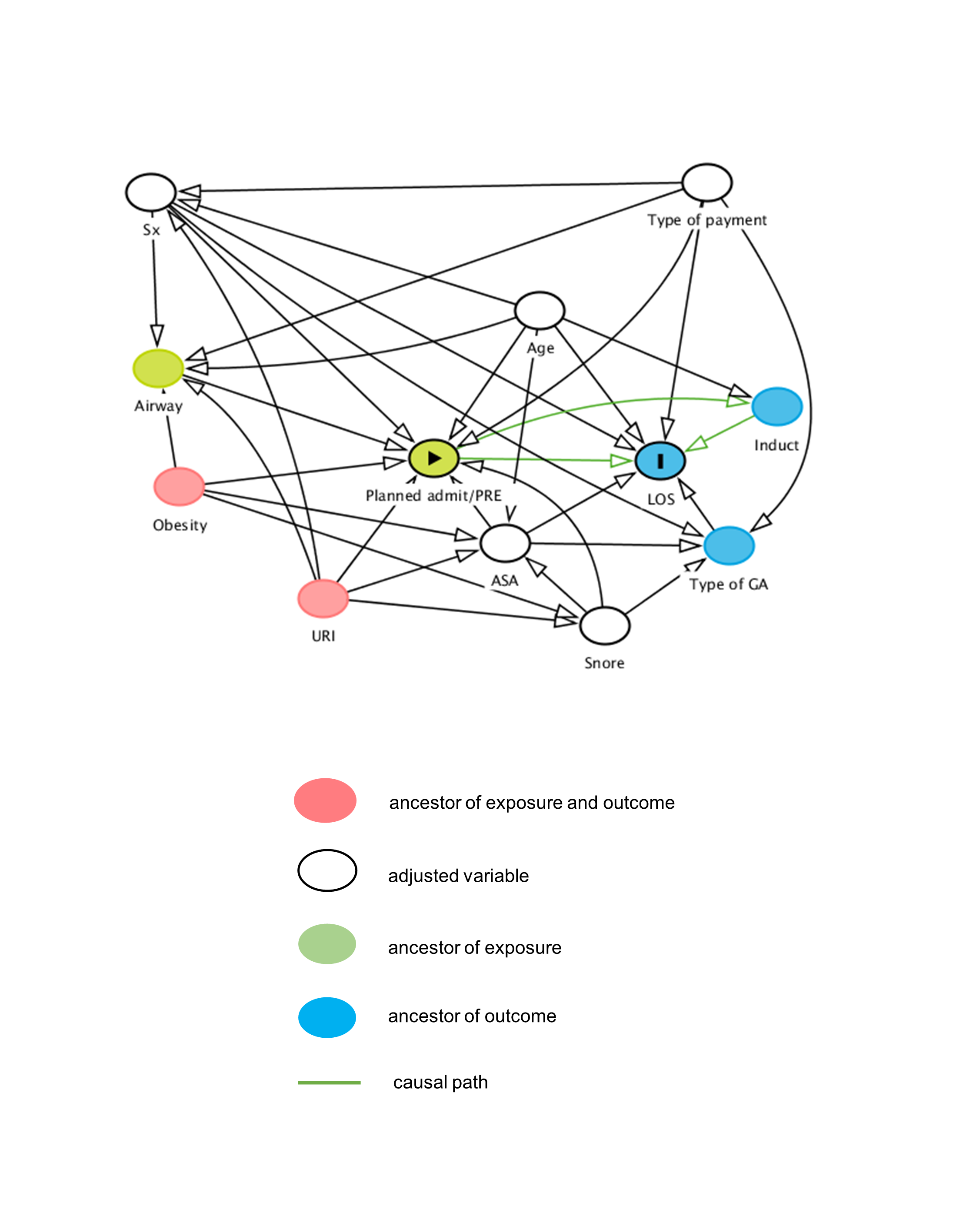

Supplement: S1 Fig — PRE, Perioperative respiratory event; LOS, Length of hospital stay; Sx, type of surgery; Airway, Airway management; ASA, American Society of Anesthesiologists; Type of GA, Type of general anesthesia; Induct, Induction agent; URI, Upper respiratory tract infection. (TIF) [file pone.0251433.s001.tif]

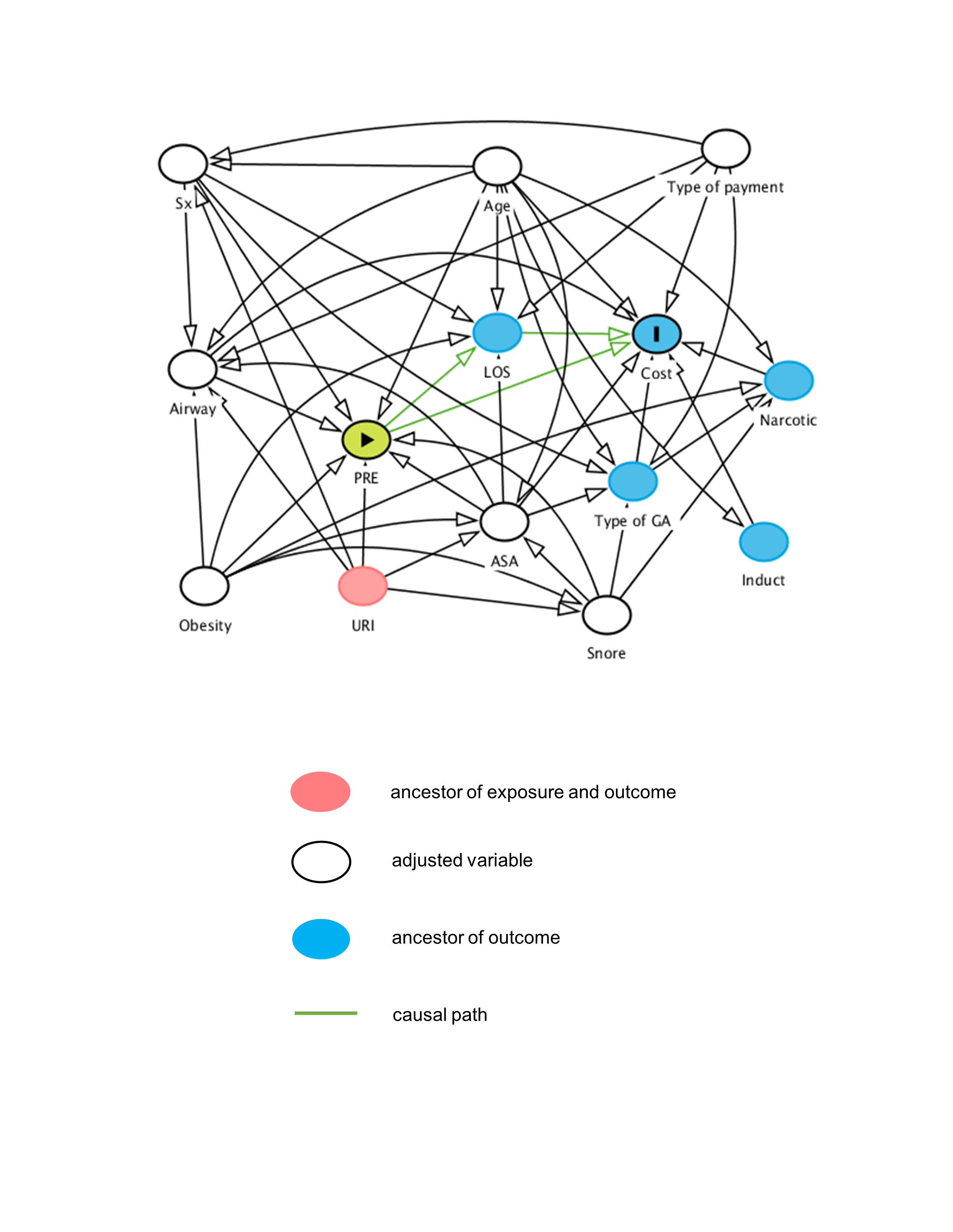

Supplement: S2 Fig — PRE, Perioperative respiratory event; LOS, Length of hospital stay; Cost, Excess hospital cost; Sx, type of surgery; Airway, Airway management; ASA, American Society of Anesthesiologists; Type of GA, Type of general anesthesia; Induct, Induction agent; URI, Upper respiratory tract infection. (TIF) [file pone.0251433.s002.tif]
