# Supplementary material for: Does perioperative respiratory event increase length of hospital stay and hospital cost in pediatric ambulatory surgery?
Source: PLoS One. 2021 May 13;16(5):e0251433. doi: 10.1371/journal.pone.0251433 (PMC8118274; doi:10.1371/journal.pone.0251433)
Supplement: Attachment — Submitted filename: response to reviewer ver 4.docx [file pone.0251433.s004.docx]

**Department of Anesthesiology**

Faculty of Medicine,

Prince of Songkla University, Hat Yai

Songkhla province, 90110 THAILAND

Tel. +66 74 429621

Fax +66 74 212900, 212903

E-mail: oomaliwa@gmail.com

15 April 2021

Dear PLOS ONE Editor,

We would like to resubmit a manuscript entitled “**Does Perioperative Respiratory Event Increase Length of Hospital Stay and Hospital Cost in Pediatric Ambulatory Surgery?**

” as an original article in your journal.

We are thankful to have a chance to revise our manuscript. To address the editor’s comment, we have deposited our protocol in [protocols.io](http://protocols.io/). We have also highlighted the changes in the current version. Attached is a point-by-point response to the reviewer’s concerns.

Reviewers' comments:

Comments to the Author

Please use the space provided to explain your answers to the questions above. You may also include additional comments for the author, including concerns about dual publication, research ethics, or publication ethics. (Please upload your review as an attachment if it exceeds 20,000 characters)

Reviewer #1: The authors present a subgroup analysis of a single-institution, 13-month prospective cohort study of the effects of a perioperative respiratory event (PRE) on length of stay and hospital cost, in children having ambulatory surgery. Of note, the authors' ref. [7] is their prior study that focused mostly on inpatients, and they state that the purpose of this manuscript's subgroup analysis is to investigate these effects on ambulatory pediatric surgery patients.

The authors employ directed acyclic graphs to determine which variables to include in their multivariable regression models. They then find that PREs with unplanned admission were associated with an increased length of stay and increased hospital cost.

The paper is a reasonable extension of the authors' prior work. There are some issues that should be addressed before publication:

1. The authors' explanation of the calculation of the contribution margin (lines 126-140) is not clear. It seems that they use the equation

(contribution margin) = 0.6 x (hospital charges)

based on assumptions from their prior work. This discussion should be clarified, as this calculation seems to be critical to one of the main conclusions - that increased costs to patients were associated with PRE.

**Response**: Thank you for your comments. It is correct that contribution margin was simply calculated from 0.6 x hospital charges, in our setting since we used a cost-to-charge ratio of 0.4 based on our previous study as well as omitting the fixed cost. We added this information in the Financial information subsection of the Methods section (line 139-140, page 7).

2. Why are there so few general surgery procedures reported? The procedures in Table 4 include ophthalmology, otolaryngology, urology and gastroenterology procedures. While excision of superficial mass and hydrocelectomy (which often includes inguinal hernia repair) are included, the authors should comment on this. I would expect there to be ambulatory hernia repairs, cholecystectomies, gastrostomy placements, and the like.

**Response**: Hernia repair (herniotomy) was grouped in the minor urologic surgery category. We added this information in the legend of Table 4. We do not have ambulatory cholecystectomies in children, usually they are adult cases and they are usually admitted (inpatients) before surgery because of fever and abdominal pain. Gastrostomy placements patients in our setting are mostly cerebral palsy patients that are usually inpatients.

3. The results from this study should be compared in more detail to those of the authors' ref. [7] in the Discussion.

**Response**: We have added this information as suggested in the discussion (line 328-332, page 24, line 334-338, 342-345, page 25).

4. The manuscript needs to be proofread again.

**Response**: The manuscript has been proofread by an English native speaker (Asst. Prof. Edward McNeil).

Reviewer #2: It is an interesting paper that provides information on little-studied aspects of ambulatory surgery in children.

**Response**: Thank you

I am struck by the fact that 27.1% of the patients were eliminated for not being able to contact their parents/guardians. Seems too high for ambulatory procedures where children get to the hospital accompanied by someone.

**Response**: Since it was a prospective cohort study, we could not obtain written informed consent from all outpatients at a given time, so the study was confined to consenting parents.

It would be convenient for the authors to explain their thinking about the relationship between PRE and the ASA classification of patients.

**Response**: Even though Table 2 shows significant difference of ASA classification between PRE and non-PRE group, the ASA classification 3 was similar between the PRE (4.8%) and non-PRE group (5.6%). A higher ASA classification could easily develop PRE; therefore, ASA classification was one of the adjusted variables in both prolonged admission and excess cost models. We add ASA detail in the Results section (line 209-212, page 11)

Line 217 has some writing problems.

**Response**: Thank you. We have rewritten it as “Approximately 40% of children in the PRE group were admitted for at least 1 day”. (line 217-218, page 11)

On line 332 the authors write about the excess hospital cost. Here, it seems to me that the variables medications and oxygen therapy should not be included in the paragraph because both variables did'n reach statistical significance.

**Response**: Thank you. We have rewritten the sentence in the discussion (line 342-345, page 25).

Statistically it has some tables that are difficult for the regular reader to understand, but they are finally understood with the specifications of each table.

**Response**: I apologize for that. I have added definitions of the specific terms in the manuscript (line 139-140, page 7, line 265-266, page 16) and in the legend of Table 4 (contribution margin) and Table 5 (geometric mean).

Reviewer #3: This is an important paper despite no surprising findings. I have almost none criticism; the number of participants seems short to overcome the effect of so many covariables.

**Response**: Thank you. We calculated the sample size to make sure the number of subjects in the study were adequate to reach our hypothesis.

Try to use more accessible and simpler language.

**Response**: We apologize for that. We have added some definitions of specific terms in the manuscript which hopefully makes it easier to read.

What is the explanation for a lower odds ratio in case of unplanned admission and non-PRE?

**Response**: Among non-PRE, preoperative unplanned admission decreased the risk of prolonged hospital stay compared to preoperative planned admission, which is basically true. If there were no perioperative complications occurring among preoperative unplanned admissions, they can be discharged home after surgery. We added this information in the discussion (line 365-368, page 26).

The inclusion of "planned admission" may question the external validity of the conclusions. Is it possible to apply the same methodology excluding "planned" admissions", a more ubiquitous reality in pediatric ambulatory surgical centers?

**Response**: Excluding preoperative planned admission out of the analysis process may be the ideal option. However, since we used secondary data with a limited sample size, we included an interaction term between type of admission and PRE, which is an alternative solution to reduce the type of admission bias.

Should you have more comments, please don’t hesitate to contact us

Thank you for your attention to our paper

Sincerely yours,

Assoc. Prof. Maliwan Oofuvong

Alan Frederick Geater

Prof. Virasakdi Chongsuvivatwong

Assoc. Prof. Thavat Chanchayanon

Bussarin Sriyanaluk

Boonthida Suwanrat

Kanjana Nuanjun
